# Multi-hop clustering routing protocol design based on simultaneous wireless information and power transfer technology and imperfect spectrum sensing for EH-CRSNs

**DOI:** 10.1038/s41598-024-57111-0

**Published:** 2024-03-20

**Authors:** Jihong Wang, Zixiao Xie, Chang Liu

**Affiliations:** School of Electrical Engineering, Northeast Electric Power University, Jilin, 132012 China

**Keywords:** Energy harvesting, Information technology

## Abstract

Existing clustering routing protocols for multi-hop energy harvesting-cognitive radio sensor networks (EH-CRSNs) generally assume perfect spectrum sensing, which is not aligned with the practical spectrum sensing capabilities of nodes in real networks. Additionally, the severe imbalance in residual energy among cluster heads (CHs) negatively affects the successful data delivery. To resolve these problems, this paper introduces a simultaneous wireless information and power transfer (SWIPT)- and imperfect spectrum sensing-based multi-hop clustering routing protocol (ES-ISSMCRP). ES-ISSMCRP makes full use of downlink EH and intra-cluster SWIPT technologies to replenish and equalize the remaining energy among nodes, further extending network lifespan while maintaining network surveillance capabilities. Specifically, to reduce the adverse impact of imperfect spectrum sensing on network performance and improve energy utilization, this paper proposes an EH-based energy level function and associated selection criteria for CHs and relays, facilitating distributed cluster formation and multi-hop routing selection between clusters. To equalize the residual energy among nodes within a cluster, ES-ISSMCRP protocol enables cluster members (CMs) to decide whether employ SWIPT technology with a power splitting (PS) receiver architecture to transmit energy to their CH while sending data. The actual energy value transmitted by CMs using SWIPT technology is deduced by calculating the PS ratio and the expected energy expenditure of nodes for data transmission. Simulation results show that ES-ISSMCRP protocol offers significant improvements over other comparative protocols in terms of extending network lifespan and enhancing network surveillance capabilities.

## Introduction

Cognitive radio sensor networks (CRSNs) are the result of the intelligent fusion of cognitive radio (CR) technology with wireless sensor networks (WSNs)^1^, where CR technology permits nodes to intermittently access unoccupied frequency bands designated for primary users (PUs) by sensing the radio spectrum environment, effectively boosting the spectrum usage and improving the performance of WSNs^2^. CRSNs inherit the architectural framework of WSNs. Their routing protocols are divided into flat routing protocols and hierarchical routing protocols, with the latter also known as clustering routing protocols. In clustering routing protocols, nodes are organized into clusters, with each cluster electing a cluster head (CH) and the rest functioning as cluster members (CMs)^3^. Data travels from CMs to the CH and then is transmitted to the sink^4^. Compared to flat routing, clustering routing offers enhanced scalability and connectivity, reduces data transmission delay and energy costs, and minimizes conflicts with PUs by carefully selecting cluster channels^5,6^, making it a prominent area of academic research.

Current clustering routing protocols in CRSNs typically rely on the assumption of perfect spectrum perception. Although this approach eases the design of protocols, it does not accurately reflect the sensing performance of nodes in practical scenarios. Due to factors like low signal-to-noise ratio and multi-path fading, nodes may face deficiencies in sensing accuracy, resulting in false alarms and missed detections^7^. Considering PUs, increased spectrum perception accuracy by CRSNs nodes implies a diminished chance of interference, which is beneficial for the regular communication of PUs. For CRSNs nodes, a lower probability of false alarms increases their chances of communicating via idle licensed frequency bands. Hence, maintaining high detection probability and low false alarm probability is essential for improving the overall system performance.

CRSNs nodes expend additional energy for spectrum sensing and switching, further draining their already limited energy resources and leading to node failure once depleted^8^. Energy harvesting (EH) is a cutting-edge solution for overcoming the energy constraints of CRSNs nodes^9^. Contrasting with conventional battery-powered devices, EH technology permits extracting energy from radio frequency (RF) signals, allowing nodes to function continuously without replacing batteries^10,11^. Consequently, the intelligent combination of EH and CRSNs, forming EH-CRSNs, has become a current research focus^12^. However, this leads to an energy disparity among nodes, as those nearer to the RF source gather more energy than those further away, creating energy imbalances and potentially causing energy holes and network segmentation. Simultaneous wireless information and power transfer (SWIPT) technology is an innovative solution for addressing the issue of energy holes in multi-hop networks, allowing for the simultaneous transmission of signals and energy, i.e., supplying power to wireless devices while interacting with them.

To reduce the adverse impact of imperfect spectrum sensing on network performance and to evenly distribute the remaining energy, thus prolonging network lifespan, this study proposes a novel SWIPT- and imperfect spectrum sensing-based muti-hop clustering routing protocol for EH-CRSNs (ES-ISSMCRP). This protocol makes full use of downlink EH and SWIPT technologies to boost and even out the remaining energy among nodes. It focuses on extending the network lifetime while preserving its surveillance capability. The innovations of this paper are summarized as follows:

- In this study, an EH-based energy level function is proposed, which comprehensively accounts for the probabilities of channel being idle or busy, along with the probabilities of false alarms and missed detections. The selection criteria for CHs and relays are designed by integrating dynamic energy arrivals and channel availability, aimed at mitigating the adverse effects of imperfect spectrum sensing on network performance and improving energy efficiency. This facilitates the implementation of distributed clustering and the establishment of multi-hop routing between clusters.
- In ES-ISSMCRP protocol, downlink EH and intra-cluster SWIPT technologies are utilized to effectively supplement and equalize the remaining energy across nodes. Intra-cluster SWIPT allows CMs to decide whether to transfer energy to their CH while transmitting data. This paper derives the actual energy values transmitted by calculating the power splitting (PS) ratio used in SWIPT technology with a PS receiver architecture and the expected energy consumption for node data transmission, thereby ensuring the practical usability of intra-cluster SWIPT technology. Simulation results indicate that ES-ISSMCRP can extend the network lifespan while ensuring high network surveillance performance.

## Related works

Clustering routing protocols for CRSNs can be categorized based on the use of EH technology for battery replenishment, distinguishing them as clustering routing protocols for non-EH-CRSNs and EH-CRSNs.

### Research on non-EH-CRSNs

Among the existing non-EH-CRSNs clustering routing protocols, based on the cluster establishment method, they are divided into single-hop and multi-hop clustering routing protocols. Single-hop protocols necessitate that all CRSNs nodes or CHs have the capability to reach the sink in a single hop, and these protocols are further subclassified into centralized and distributed clustering protocols. Centralized protocols including CogLEACH-C^13^, Fuzzy C-means^14^, and IMOCRP^15^ utilize CHs to reduce energy use and improve data efficiency while simplifying network administration, though they are limited by potential single points of failure and communication bottlenecks, which can hamper network scalability. Specifically, nodes in CogLEACH-C protocol communicate their perceived available channel count, remaining energy level, and location within the network to the sink, and then the sink selects the most suitable CHs. Fuzzy C-means protocol divides the network into several clusters with the goal of minimizing the cumulative squared distance between member nodes and the cluster center. It chooses the best CHs based on various parameters. IMOCRP protocol automatically identifies the best CHs and the ideal number of clusters, focusing on minimizing node energy use and equitably distributing residual energy among nodes. Distributed clustering protocols like CogLEACH^16^, NSAC^17^, and WCM^18^, allow for more flexible CHs selection based on node energy levels, thus enhancing reliability and scalability, reducing bottlenecks and failures, and enabling more even energy consumption distribution across the network. Specifically, nodes in CogLEACH protocol randomly decide whether they can become CHs based on their own weights, and the higher the number of available free channels, the higher the possibility of nodes to become CHs. NSAC protocol sets the weights for the nodes based on the node's remaining energy and the node's available channels and selects the nodes with the highest weights as the CHs. WCM achieves optimal clustering by solving optimization problems, selecting CHs and CMs based on temporal-spatial correlation, confidence levels, and residual energy, while minimizing energy consumption by limiting spectrum sensing to CHs.

In single-hop CRSNs, CHs that are further away from the sink need to expend more energy to transmit data directly to the sink in one hop, resulting in premature node death and significantly compromising the network surveillance capability. Multi-hop clustering routing protocols alleviate this by resolving routing issues between clusters, enabling CRSNs nodes that are incapable of reaching the sink in a single hop to send data packets to the sink through relay nodes. Such protocols are further subdivided into uniform and uneven clustering protocols. In uniform clustering protocols, all CRSNs nodes maintain the same cluster radius, i.e., the distance for information exchange with neighboring nodes during CHs selection and cluster construction, as is the case with protocols like DSAC^19^, EACRP^20^, and SACR^21^.

In uniform clustering protocols, CHs situated near the sink bear the responsibility of receiving and aggregating intra-cluster data as well as aiding in relaying data from outer clusters, leading to quicker energy depletion and potential energy holes or network fragmentation. To address this, uneven clustering protocols that adaptively adjust cluster radius sizes have been proposed. Such protocols divide the network into clusters of varying sizes, with smaller-radius clusters located closer to the sink and larger-radius clusters situated farther away. In terms of cluster radius calculation and CHs selection, LEAUCH^22^ protocol uses the number of vacant channels to determine candidate CHs, then calculates the cluster radius based on Euclidean distance to the sink and selects nodes with high residual energy within the cluster radius as final CHs. R-bUCRP^23^ protocol calculates cluster radii similarly to LEAUCH but identifies candidate CHs by comparing a random number that lies between 0 and 1 with a predetermined threshold. Final CHs are selected based on a weighted sum of node reputation and residual energy. OACUCAPTEEN^24^ refines cluster radius calculation and CHs selection based on node residual energy. Building upon LEAUCH protocol, ESAUC^25^ redefines cluster radius calculation by incorporating factors like remaining energy, number of neighboring nodes and vacant channels and selects nodes with the maximum weighted sum of residual energy and available channels as final CHs. Our previous work ISSMCRP^26^ accounts for the influence of sensing errors on network performance. ISSMCRP calculates the detection level function for available channels and selects nodes with strong detection capability, high remaining energy, and large number of neighbors as CHs. EBUCRP^27^ derives the optimal cluster radius probabilistically to minimize network energy consumption and equalize the residual energy levels of nodes. Nodes with high residual energy, numerous available idle channels, and low communication energy consumption with neighboring nodes are selected as CHs. While uneven clustering protocols have successfully tackled issues related to energy holes and hot spot, they have not yet rectified the fundamental limitation of energy scarcity in sensor nodes.

### Research on EH-CRSNs

Clustering routing protocols in EH-CRSNs effectively enhances the network's energy utilization by exploiting EH technology, consequently extending its lifespan. The associated strategies and mechanisms of these protocols are developed to provide substantial support and guarantees for the practical application of EH-CRSNs. The multi-hop clustering routing protocol RFMCRP^8^ for RF EH-CRSNs introduces an energy control strategy for the management of node states. This approach aims to mitigate performance degradation due to energy insufficiency. It theoretically derives the optimal cluster count with the objective of minimizing the total energy consumed in data transmission. The protocol selects nodes with high energy function levels within the communication range, more common available channels with neighboring nodes, and numerous neighbors as CHs. Reference^28^ applies SWIPT technology based on the PS receiver architecture to CRSNs nodes. This work derives an expression for the outage probability of PUs under Nakagami-m fading channels and utilizes this expression to study the throughput of the primary system. References^29,30^ apply the SWIPT technology based on the time switching (TS) receiver architecture to CRSNs nodes. Specifically, reference^29^ derives the expressions for the outage probabilities and network throughput of both the primary and secondary systems under Nakagami-m fading channels. It then studies the system performance based on these derived expressions. Reference^30^ further derives expressions for the outage probability, asymptotic outage probability, system throughput, and energy efficiency of both primary and secondary systems under Nakagami-m fading channels. This work further explores their ergodic capacity and asymptotic ergodic capacity. In reference^31^, research is conducted on the trade-off between rate and energy in systems employing SWIPT technology under 2 different non-linear EH models. Current research in EH-CRSNs, including RFMCRP protocol, does not integrate SWIPT to tackle the energy hole problem. Studies related to SWIPT in the aforementioned literature predominantly concentrate on analyzing system performance, rather than on utilizing clustering protocols for logical node grouping and joint operations to diminish node energy consumption. Consequently, there is an urgent need to develop a novel clustering routing protocol for EH-CRSNs that utilizes SWIPT to properly balance the residual energy among nodes in each cluster of EH-CRSNs. The characteristic analysis and comparison of the aforementioned clustering routing protocols is shown in Table 1.

**Table 1:** Comparison of the characteristics of existing clustering routing protocols.

References	Target network	Protocol type	Considering control overhead	Applying SWIPT	Considering imperfect spectrum sensing	Single-hop/multi-hop
^13,14^	Non-EH-CRSNs	Uniform (centralized)	×	×	×	Single-hop
^15^	Non-EH-CRSNs	Uniform (centralized)	√	×	×	Single-hop
^16^	Non-EH-CRSNs	Uniform (distributed)	×	×	×	Single-hop
^17^	Non-EH-CRSNs	Uniform (distributed)	×	×	×	Single-hop
^18^	Non-EH-CRSNs	Uniform (hybrid)	×	×	×	Single-hop
^19–21^	Non-EH-CRSNs	Uniform (distributed)	×	×	×	Multi-hop
^22^	Non-EH-CRSNs	Uneven	×	×	×	Multi-hop
^23–25^	Non-EH-CRSNs	Uneven	√	×	×	Multi-hop
^26^	Non-EH-CRSNs	Uneven	√	×	√	Multi-hop
^27^	Non-EH-CRSNs	Uneven	√	×	×	Multi-hop
^8^	EH-CRSNs	Uneven	√	×	×	Multi-hop
^28–31^	EH-CRSNs	–	×	√	×	−
Ours	EH-CRSNs	Uneven	√	√	√	Multi-hop

## System model

The sink is located at the center of a circular network with a radius of *R*, where *N* identical CRSNs nodes and *M* PUs are evenly and randomly scattered. Each CRSNs node has a unique ID, and its location will not change once deployed. The network is segmented into *z* concentric layers, each with a width of *R*_*t*_, and the layers are sequentially arranged from inside to outside as layer 1, 2, …, *z*. In this study, semi-Markov ON/OFF model^32^ is utilized to replicate the dynamic spectrum usage patterns of PUs. Under this model, PUs alternately change between busy/idle states on authorized channels, with the duration of each state being an independent random variable. It is assumed that the ambient noise is characterized by zero-mean, $\delta_{n}^{2}$-variance Gaussian white noise. The signal of PU_*j*_ is characterized as an independent and identically distributed random process with zero mean and variance of $\delta_{x,j}^{2}$. For CRSNs node *i* employing energy detection^33^, the probabilities of false alarm *P*_*f*(*i*,*j*)_ and detection *P*_*d*(*i*,*j*)_ for detecting PU_*j*_ are given as^34^:

1 $$P_{f(i,j)} = Q\left( {\frac{{\lambda - \delta_{n}^{2} }}{{\delta_{n}^{2} /\sqrt {Z/2} }}} \right)$$

2 $$P_{d(i,j)} = Q\left( {\frac{{\lambda - (\delta_{n}^{2} + \delta_{x(i,j)}^{2} )}}{{(\delta_{n}^{2} + \delta_{x(i,j)}^{2} )/\sqrt {Z/2} }}} \right)$$

where *Q*(·) signifies the Q function; *Z* denotes the count of sampling points, calculated as *Z* = *T*_*s*_ × *f*_*s*_ (*T*_*s*_ being the spectrum perception duration and *f*_*s*_ the sampling frequency); *λ* represents the energy detection threshold, and when *P*_*f*(*i*,*j*)_ is a predetermined small value $\overline {P}_{f(i,j)}$, *λ* is derived from Eq. (1) following the Neyman-Pearson criterion^35^, as illustrated in Eq. (3); $\delta_{x(i,j)}^{2}$ stands for the signal power of PU_*j*_ received by node *i*, as indicated in Eq. (4). *d*_*to*PU(*i*,*j*)_ refers to the Euclidean distance between node *i* and PU_*j*_. If node *i* is located within PU_*j*_'s interference protection range (IPR), $\delta_{x(i,j)}^{2}$ is determined as $\delta_{x(i,j)}^{2} = \left| {h_{(i,j)} } \right|\delta_{x,j}^{2}$; *h*_(*i*,*j*)_ denotes the channel gain between node *i* and PU_*j*_, calculable through Eq. (5)^36^; Otherwise, the received signal power of PU_*j*_ at node *i* is considerably weak and can be negligible.

3 $$\lambda = \delta_{n}^{2} \times (Q^{ - 1} (\overline{P}_{f(i,j)} )/\sqrt {Z/2} + 1)$$

4 $$\delta_{x(i,j)}^{2} \left\{ \begin{gathered} = \left| {h_{(i,j)} } \right|\delta_{x,j}^{2} {\text{ if}}\, \, d_{{to{\text{PU(}}i{,}j{)}}} \le {\text{IPR}} \hfill \\ \approx {0}\quad \quad \quad \quad \,{\text{otherwise}} \hfill \\ \end{gathered} \right.\,$$

5 $$h_{{{(}i,j{)}}} = \left\{ {\begin{array}{*{20}c} {\frac{{G_{T} G_{R} l^{2} }}{{16{\uppi }^{2} d_{{to{\text{PU(}}i,j{)}}}^{2} }}\quad {\text{if }}d_{{to{\text{PU(}}i,j{)}}} \le d_{0} } \\ {\frac{{G_{T} G_{R} h_{T}^{2} h_{R}^{2} }}{{d_{{to{\text{PU(}}i,j{)}}}^{4} }}\quad {\text{otherwise }}} \\ \end{array} } \right.$$

Equation (5) indicates that if *d*_*to*PU(*i*,*j*)_ is equal to or less than the distance threshold *d*_0_, the PU signal attenuation adheres to the free-space path loss model; if it exceeds *d*_0_, it follows the multi-path fading loss model. *G*_*T*_ and *G*_*R*_ are the respective gains of the transmitting and receiving antennas, and *l* represents the wavelength of the signal emitted by PUs. *h*_*T*_ and *h*_*R*_ refer to the heights of the transmitting and receiving antennas, respectively.

Similarly, in downlink RF EH, the energy that CRSNs node *i* can harvest relies on the transmit power *P*_*sink*_ of the RF energy source, i.e., the sink, the wavelength of the RF signal *γ*, the Euclidean distance *d*_*i,sink*_ between the RF energy source and the EH node, and the duration of the EH process *t*_*EH*_, as shown in Eq. (6).

6 $$E_{in} \left( i \right) = \left\{ \begin{gathered} f\left( {P_{sink} \times \frac{{G_{T} G_{R} \gamma^{2} }}{{16{\uppi }^{2} d_{i,sink}^{2} }}} \right) \times t_{EH} \, if \, d_{i,sink} \le d_{0} \hfill \\ f\left( {P_{sink} \times \frac{{G_{T} G_{R} h_{T}^{2} h_{R}^{2} }}{{d_{i,sink}^{4} }}} \right) \times t_{EH} \, otherwise \hfill \\ \end{gathered} \right.$$

where *f*(*x*) refers to the non-linear EH model discussed in reference^37^.

The energy consumption model presented in reference^38^ is leveraged to compute the energy expenditure for information transmission. The energy consumption expression for a source node to send *L* bits of information to its desired destination located at a distance *d* is depicted as Eq. (7):

7 $$E_{trans} \left( {L{, }d} \right){ = }\left\{ \begin{gathered} L \times E_{elec} + L \times E_{fs} \times d^{2} {\text{ if}}\,d \le d_{0} \hfill \\ L \times E_{elec} + L \times E_{mp} \times d^{4} {\text{ otherwise}} \hfill \\ \end{gathered} \right.$$

where *E*_*elec*_ represents the energy consumed by electronic circuitry to send/receive a single bit of data; Under the assumption that the signal transmission adheres to the free-space path loss model, *E*_*fs*_ is the power amplifier's energy consumption per bit under this model, and *E*_*mp*_ denotes that under multi-path fading loss model.

If the information transmission rate is *R*_*b*_, then the energy used by the amplifier to transfer *L* bits of data, *E*_*TX*_(*L*,*d*), is the product of the node transmit power *P*_*T*_ and the inverse of the information transmission rate, as indicated in Eq. (8):

8 $$E_{TX} \left( {L{, }d} \right) = L \times P_{T} \times \frac{1}{{R_{b} }}$$

Incorporating Eq. (7) into Eq. (8) and simplifying yields Eq. (9):

9 $$P_{T} = \left\{ \begin{gathered} E_{fs} \times R_{b} \times d^{2} {\text{ if}}\,d \le d_{0} \hfill \\ E_{mp} \times R_{b} \times d^{4} {\text{ otherwise}} \hfill \\ \end{gathered} \right.$$

In this case, the power threshold *P*_*thresh*_ required at the receiving end for successful information decoding is defined as:

10 $$P_{thresh} = \left\{ \begin{gathered} \frac{{E_{fs} R_{b} G_{T} G_{R} \gamma^{2} }}{{16{\uppi }^{2} }}{\text{ if}}\,d \le d_{0} \hfill \\ E_{mp} R_{b} G_{T} G_{R} h_{T}^{2} h_{R}^{2} {\text{ otherwise}} \hfill \\ \end{gathered} \right.$$

Based on the findings in reference^38^, with *E*_*fs*_ being 10 pJ/bit/m^2^ and *E*_*mp*_ at 0.0013 pJ/bit/m^4^, it is inferred from Eq. (10) that *P*_*thresh*_ should be no less than 6.8 nW for successful decoding of received data.

## SWIPT and imperfect spectrum sensing-based multi-hop clustering routing protocol for EH-CRSNs

As an advancement of ISSMCRP protocol, ES-ISSMCRP protocol is specifically designed for time-triggered traffic, and it focuses on efficiently replenishing and equalizing the remaining energy of CRSNs nodes under the premise of mitigating the adverse impacts of imperfect spectrum sensing on network performance, consequently prolonging the network lifetime. ES-ISSMCRP protocol operates in 5 distinct stages: spectrum sensing, non-linear EH, CHs election and cluster formation, inter-cluster route establishment, and data transmission. In each round, each EH-CRSNs node updates and saves details about itself and its adjacent nodes, and autonomously establishes clusters and develops multi-hop routing based on the gathered information.

### CHs election and cluster formation stage

In ES-ISSMCRP protocol, every node in the first layer forms its own cluster, thereby saving the energy that would be used for selecting CHs and building clusters, and preserving energy for relaying packets of nodes in other layers situated farther from the sink. Node *i* beyond the first layer determines its cluster radius *R*_*c*_[*l*(*i*)] by using Eq. (18) in reference^26^. Here, *l*(*i*) represents the layer number of node *i*. Layers nearer to the sink have smaller cluster radii, which significantly lowers the energy needed by CHs in those layers for receiving and aggregating intra-cluster data. This method helps to conserve energy for the relaying data packets from nodes in outer layers, thereby achieving a balance in energy usage across the CHs. Node *i* calculates its CHs competition weight based on the available channel detection level function *P*_*CL*_(*i*), its residual energy *E*_*res*_(*i*), and the energy *E*_*in*_(*i*) that node *i* harvests from downlink, as detailed in Eq. (11). It broadcasts its information and CHs competition weight within *R*_*c*_[*l*(*i*)] and receives similar broadcasts from neighboring nodes in the same layer. ES-ISSMCRP protocol assesses whether a node beyond the first layer can become a CH by comparing the competition weights in its vicinity. If a node's competition weight is lower than its neighbors', it exits the competition; otherwise, it becomes a CH. Nodes with superior channel detection capabilities, higher potential residual energy levels, and a larger number of neighbors have a higher likelihood of becoming CHs.

11 $$EH{ - }Compt(i) = P_{CL} (i) \times (E_{res} (i) + E_{in} (i)) \times Neigh(i)$$

where *Neigh*(*i*) denotes the count of active neighboring nodes for node *i*. *P*_*CL*_(*i*) is derived by node *i*, taking into account its specific false alarm and missed detection probabilities, and the probabilities of channel being either free or busy when detection is correctly assumed, as explicitly outlined in Eq. (12).

12 $$P_{CL} (i) = \sum\limits_{{c \in {\varvec {Channel}}_{{\varvec{i}}} }} {[P_{off(i,c)} \times (1 - P_{f(i,c)} ) - P_{on(i,c)} \times (1 - P_{d(i,c)} )]}$$

where *c* ∈ ***Channel***_***i***_ denotes that channel *c* is identified as available by node *i*. $P_{{off{(}i,c{)}}} = \prod\nolimits_{j = 1}^{M} {P_{{off{(}i,j,c{)}}} }$ and $P_{{on{(}i,c{)}}} = 1 - P_{{off{(}i,c{)}}}$ refer to the probabilities that node *i* determines the licensed channel *c* as being idle and occupied, respectively, under the assumption of accurate detection. Specifically, *P*_*off*(*i*,*j*,*c*)_ and *P*_*on*(*i*,*j*,*c*)_ are the probabilities of node *i* accurately detecting whether PU_*j*_ is and is not occupying channel *c* during spectrum sensing. If *d*_*to*PU(*i*,*j*)_ ≤ *IPR*, *P*_*off*(*i*,*j*,*c*)_ = *P*_*Coff*(*j*,*c*)_ and *P*_*on*(*i*,*j*,*c*)_ = *P*_*Con*(*j*,*c*)_. Otherwise, due to the insignificantly low received signal power of PU_*j*_, node *i* will invariably judge that PU_*j*_ is not occupying the channel, thus *P*_*off*(*i*,*j*,*c*)_ = 1 and *P*_*on*(*i*,*j*,*c*)_ = 0. *P*_*Coff*(*j*,*c*)_ and *P*_*Con*(*j*,*c*)_ are the probabilities of PU_*j*_ not using and using licensed channel *c*, respectively, which are obtained from historical channel usage. The probability *P*_*off*(*i*,*c*)_ × (1 − *P*_*f*_(*i*,*c*)) reflects the likelihood of node *i* accurately detecting channel *c* as idle, while *P*_*on*(*i*,*c*)_ × (1 − *P*_*d*_(*i*,*c*)) indicates the chance of node *i* mistakenly detecting channel *c* as idle. When a channel is determined as available, the protocol subtracts the probability of incorrect detection from that of correct detection as a penalty.

CHs broadcast CHs announcements within the cluster radius. Ordinary nodes that are not yet clustered look for the CH within their own transmission range that has common available channels and the highest CHs competition weight, to which they send a join request, thereby marking themselves as clustered. CHs acknowledge these join requests from the ordinary nodes and list them as their CMs. Ordinary nodes that fail to identify a CH become CHs by default. CHs that do not receive any join requests form clusters independently. For improving the success rate of data transmission within clusters, nodes are required to identify and use the appropriate idle channel for data transmission. Hence, CMs use Eq. (13) to determine the idle detection accuracy *P*_*r*(*i*,*c*)_ for each available channel, selecting the one that is both commonly idle with the CHs and has the maximum *P*_*r*(*i*,*c*)_ as the cluster channel. CHs apply channel switching to gather data within the cluster. To reduce energy consumption by minimizing channel switching frequency, CHs consecutively schedule all members using the same channel for data transmission. After completing transmissions on one channel, they move to the next, continuing until all nodes within the cluster have transmitted their data.

13 $$P_{r(i,c)} = \frac{{P_{off(i,c)} \times (1 - P_{f(i,c)} )}}{{P_{off(i,c)} \times (1 - P_{f(i,c)} ) + P_{on(i,c)} \times (1 - P_{d(i,c)} )}}$$

### Inter-cluster route establishment and data transmission stages

In ES-ISSMCRP protocol, CHs in all layers except the first must choose suitable relay nodes based on relay competition values as indicated in Eq. (14), forming inter-cluster multi-hop routes for data packet forwarding to the sink.

14 $$EH{ - }Relaycompt\left( i \right){ = }P_{CL} \left( i \right) \times (E_{res} (i) + E_{in} (i)) \times \frac{1}{{E_{elec} + E_{fs} \times d_{i,sink}^{2} }}$$

where $E_{elec} + E_{fs} \times d_{i,sink}^{2}$ denotes the energy expenditure of node *i* to transmit unit data to the sink. Node that exhibits superior channel detection capabilities, higher levels of potential remaining energy, and is closer to the sink has a greater chance of being the next hop. The routing process ceases if the relay node is in the first layer; if not, the next hop with the highest relay competition value is selected from the inner-layer CHs, which is within the transmission range, shares common idle channels, and is located closer to the sink. If no appropriate relay node is found, ES-ISSMCRP identifies a CM within the cluster that is the closest to the sink and capable of communicating with inner-layer CHs as a potential relay. If a suitable CM is found, the process of identifying the next hop relay continues until it reaches a node in the first layer. If no adequate CM is available, ES-ISSMCRP seeks for a same-layer CH within the transmission range that shares common channels, is nearer to the sink, and possesses the highest competition value. The qualified same-layer CH then becomes the next hop, and this selection process is repeated until the relay reaches a first-layer node or no further viable routing path is available.

Once the routing is finalized, each relay node chooses the channel with the greatest idle detection accuracy from the common available channels shared with its subsequent hop. This strategy is aimed at improving the successful rate of transmissions between clusters by utilizing the inter-cluster channel that is most likely to be idle.

**Figure 1:**
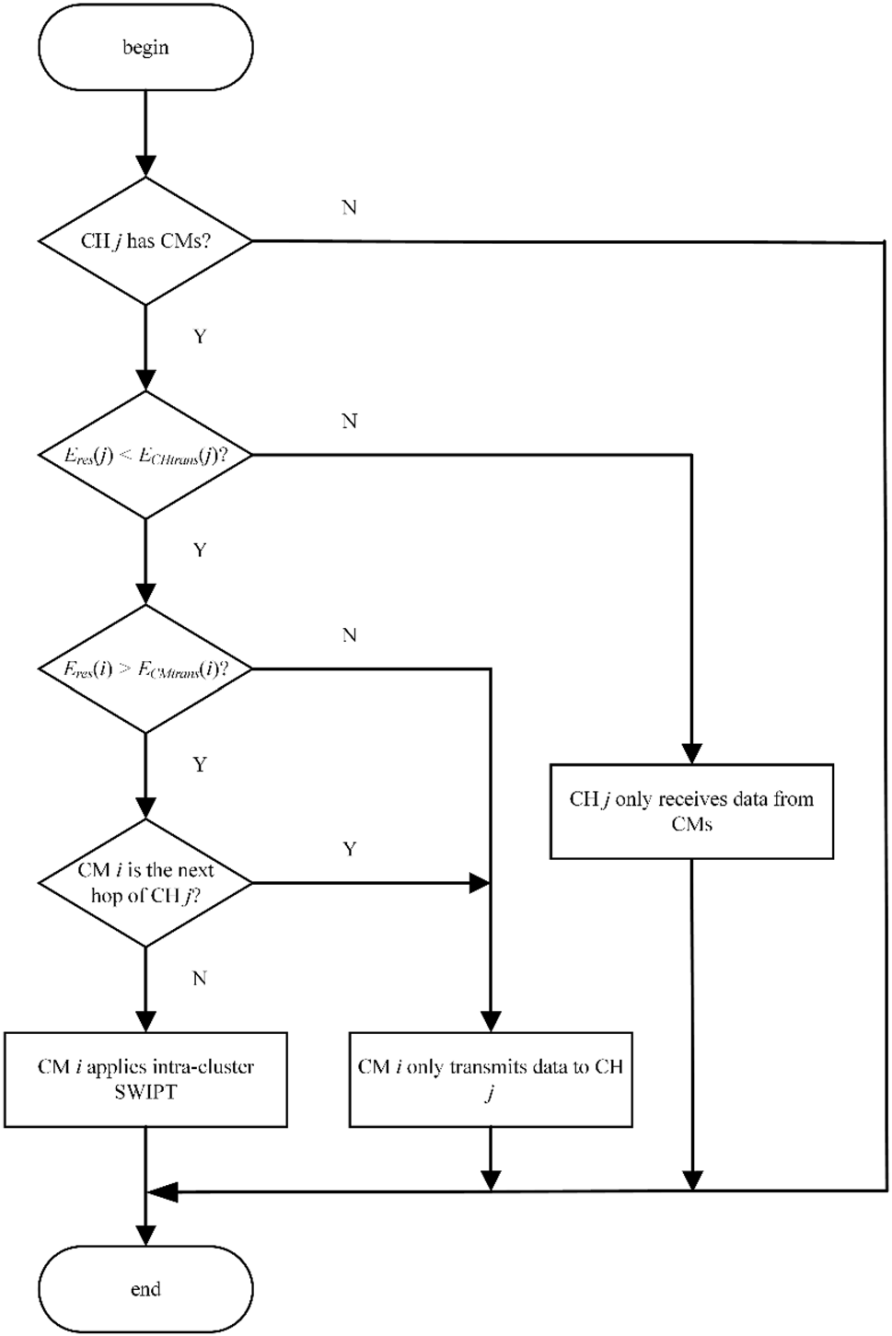
Flowchart of the intra-cluster SWIPT mechanism in ES-ISSMCRP protocol.

Data transmission encompasses both intra-cluster and inter-cluster data transfers. Intra-cluster data transfer requires CHs that have CMs to schedule time division multiple access (TDMA) time slots for their CMs. The CMs receive the schedule information and decide whether to transfer energy to the CH through PS architecture-based SWIPT while transmitting data based on their energy levels. The PS ratio *ρ* is determined by *ρ* = *P*_*thresh*_/*P*_*R*_, and *P*_*R*_ denotes the aggregate actual power that the destination node receives. Figure 1 displays the detailed process of ES-ISSMCRP protocol employing intra-cluster SWIPT mechanism.

CMs make a decision on employing SWIPT for parallel data and energy transmission within the cluster, considering their specific role, residual energy, and the CH's residual energy. (1) When the residual energy *E*_*res*_(*j*) of CH *j* falls below its anticipated energy expenditure for the data transmission *E*_*CHtrans*_(*j*), and if the residual energy of CM *i E*_*res*_(*i*) exceeds its anticipated energy expenditure *E*_*CMtrans*_(*i*) for the round, and CM *i* is not the subsequent hop for CH *j*, CM *i* will transfer all its extra energy to CH *j*. The expressions for *E*_*CHtrans*_(*j*) and *E*_*CMtrans*_(*i*) are exhibited in Eqs. (15) and (16), respectively. (2) CMs acting as relay nodes bear the burden of inter-cluster data transmission. To prevent energy loss from two-way transmission between these CMs and the CH, they do not employ SWIPT technology to supply energy to the CH.

15 $$E_{CHtrans} (j){ = }CMs\left( {{\text{CH }}j} \right) \times \left( {E_{elec} + E_{DA} } \right) \times L + \left( {E_{elec} + E_{fs} \times d_{tonext}^{2} (j)} \right) \times L$$

where *E*_*DA*_ refers to the energy used for aggregating per unit of data; *CMs*(CH *j*) indicates the count of CMs belonging to CH *j*; *d*_*tonext*_(*j*) is the Euclidean distance from CH *j* to its relay; *L* stands for the data packet length. The two components on the right-hand side of Eq. (15) correspond to the energy expended by CH *j* in receiving and aggregating intra-cluster data, and in transmitting this aggregated data to the next-hop relay.

16 $$E_{CMtrans} (i){ = }\left( {E_{elec} + E_{fs} \times d_{toCH}^{2} (i)} \right) \times L$$

where *d*_*toCH*_(*i*) signifies the Euclidean distance between CM *i* and CH *j*.

The energy replenished by CM *i* to CH *j* using SWIPT technology is given by the following expression:

17 $$E_{send} (i) = E_{res} (i) - E_{CMtrans} (i)$$

The energy harvested by destination node *j* is transformed into usable energy stored within the hybrid energy storage unit. Hence, the energy *E*_*S*_(*i*,*j*) received by CH *j* from the SWIPT-based transmission by CM *i* is calculated using Eqs. (10) and (17), as shown in Eq. (18):

18 $$E_{S} (i,j){ = }f\left( {E_{send} (i)/d_{toCH}^{2} (i) - L \times P_{thresh} \times \frac{1}{{R_{b} }}} \right)$$

where *f*(*x*) refers to the non-linear EH model discussed in reference^37^.

In the process of inter-cluster data transfer, relay nodes are burdened with increased traffic loads, which can lead to significant energy depletion and the risk of early node failure. The use of inter-cluster SWIPT technology enables CHs to simultaneously transfer energy and data to their subsequent hops, aiming to equalize energy usage across clusters. However, in large-scale EH-CRSNs where CHs of various layers are relatively distant, energy wastage occurs during energy transmission to the next hop, resulting in some resource wastage. Additionally, in the stage of cluster formation, ES-ISSMCRP protocol divides the network into uneven clusters based on the calculated cluster radius for each layer, reducing energy consumption of inner-layer nodes and reserving energy for relaying data packets from nodes in outer layers, thus effectively equalizing the energy expenditure across clusters. As a result, SWIPT technology is not applied to the inter-cluster data transfer in this paper.

## Simulation analysis and performance verification

In this paper, the MATLAB simulation tool is employed to conduct a performance evaluation of the proposed ES-ISSMCRP protocol. 450 CRSNs nodes and 50 PUs are randomly and uniformly deployed in a circular network monitoring area with a radius of *R* = 150 m, with the sink located at the center of the network. The sink consistently supplies RF energy to CRSNs nodes over a fixed time period *t*_*EH*_ = 0.2 s. Given that the maximum transmission range *R*_*t*_ of CRSNs nodes is 50 m, the entire network is divided into 3 layers, indicating that CRSNs nodes belong to either layer 1, 2, or 3. The cluster radii for layer 2 and layer 3 are determined using the theoretical derivation method in our previous work^26^. Each surviving node in every round must transmit the monitoring data collected from the environment to the sink. Additionally, while maintaining the same network radius, we increased the number of CRSNs nodes from 450 to 900 to explore the impact of node density on the performance of ES-ISSMCRP protocol, i.e., the node density has doubled compared to its original value. The additional parameters for the simulation are detailed in Table 2^8,15,22,26,27,38,39^.

**Table 2:** Simulation parameter settings.

Parameters	Values
Battery storage capacity *E*_*maxb*_	1 J
Total CRSNs nodes count *N*	450
Total PUs count *M*	50
Network radius *R*	150 m
The maximum transmission range of CRSNs nodes *R*_*t*_	50 m
Transmit power of the sink *P*_*T*_	100 W
EH duration *t*_*EH*_	0.2 s
Data packet length *L*	1024 bits
Data transmission rate *R*_*b*_	1 Mbps
Minimum received power required for successful information decoding *P*_*thresh*_	6.8 nW
Energy consumption for data aggregation per bit *E*_*DA*_	5 nJ/bit/packet

**Figure 2:**
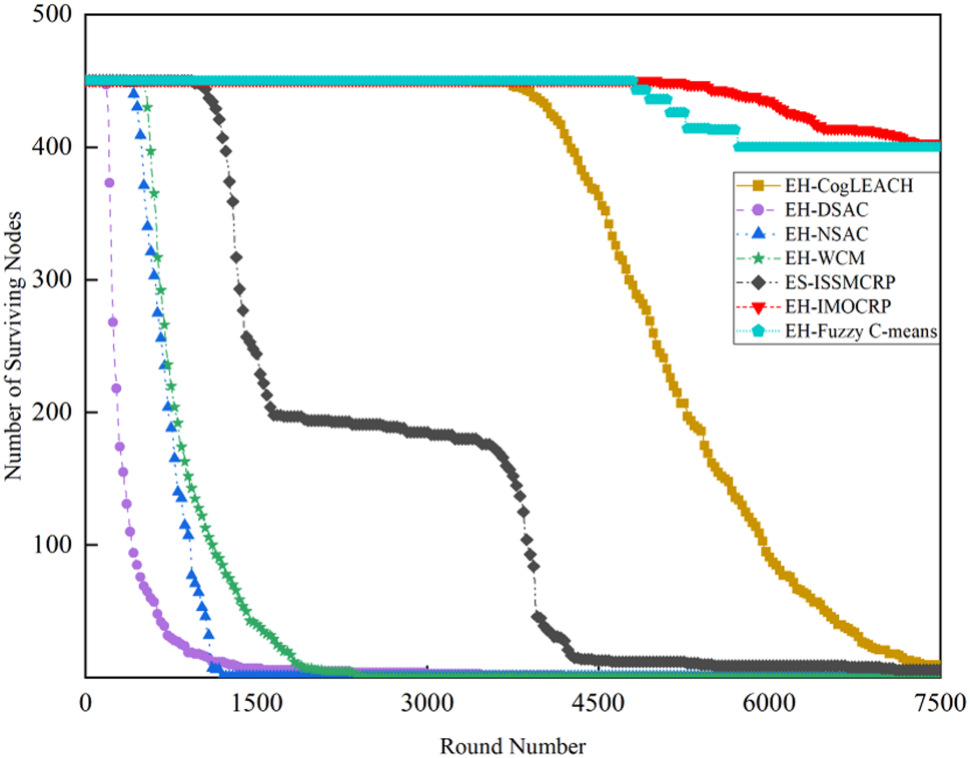
Comparison results of the number of surviving nodes across various protocols.

**Figure 3:**
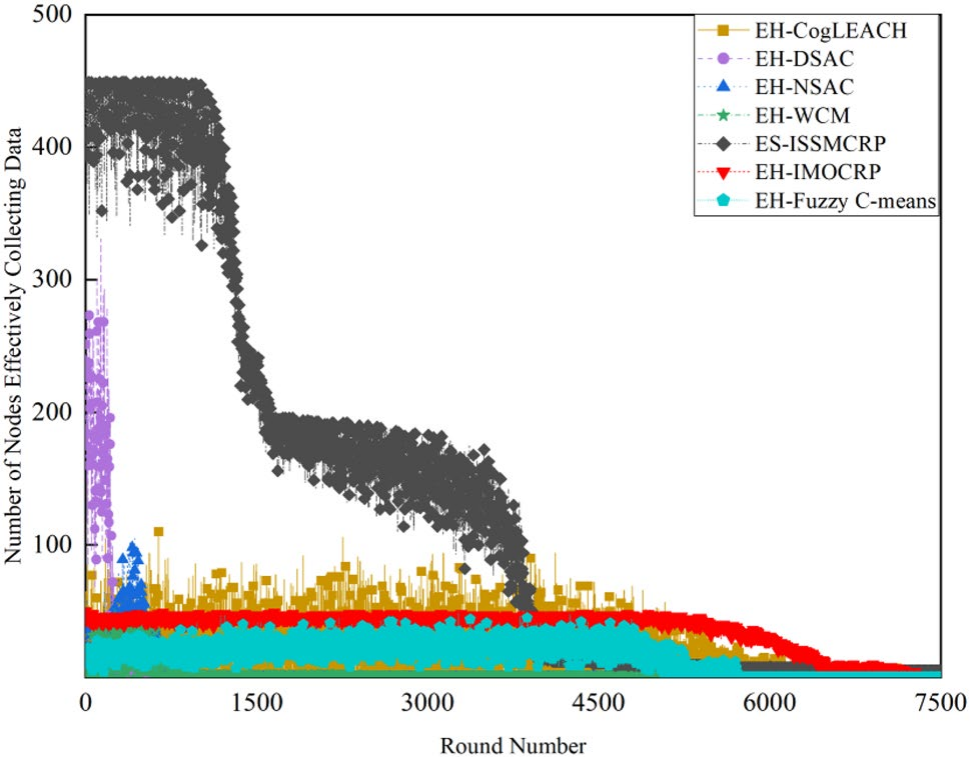
Comparison results of the number of nodes effectively collecting data across various protocols.

To demonstrate the superiority of ES-ISSMCRP protocol in extending network lifetspan and enhancing network surveillance capability, a comparative analysis is conducted with existing CRSNs clustering routing protocols, including CogLEACH^16^, DSAC^19^, NSAC^17^, WCM^18^, IMOCRP^15^, and Fuzzy C-means^14^. These protocols are inherently non-EH-CRSNs clustering protocols. To ensure a fair comparison, we incorporate a linear EH mechanism into these protocols, allowing each surviving CRSNs node to perform linear EH at the start of each round and store the collected energy in their on-board batteries. After adding EH functionality, these protocols are referred to as EH-CogLEACH, EH-DSAC, EH-NSAC, EH-WCM, EH-IMOCRP, and EH-Fuzzy C-means. The comparison in terms of the number of surviving nodes and nodes effectively collecting data in each protocol is depicted in Figs. 2 and 3.

**Figure 4:**
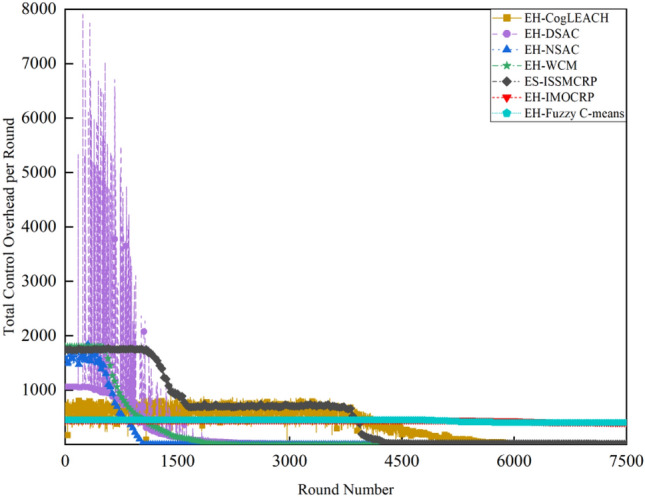
Comparison results of the total control overhead per round across various protocols.

**Figure 5:**
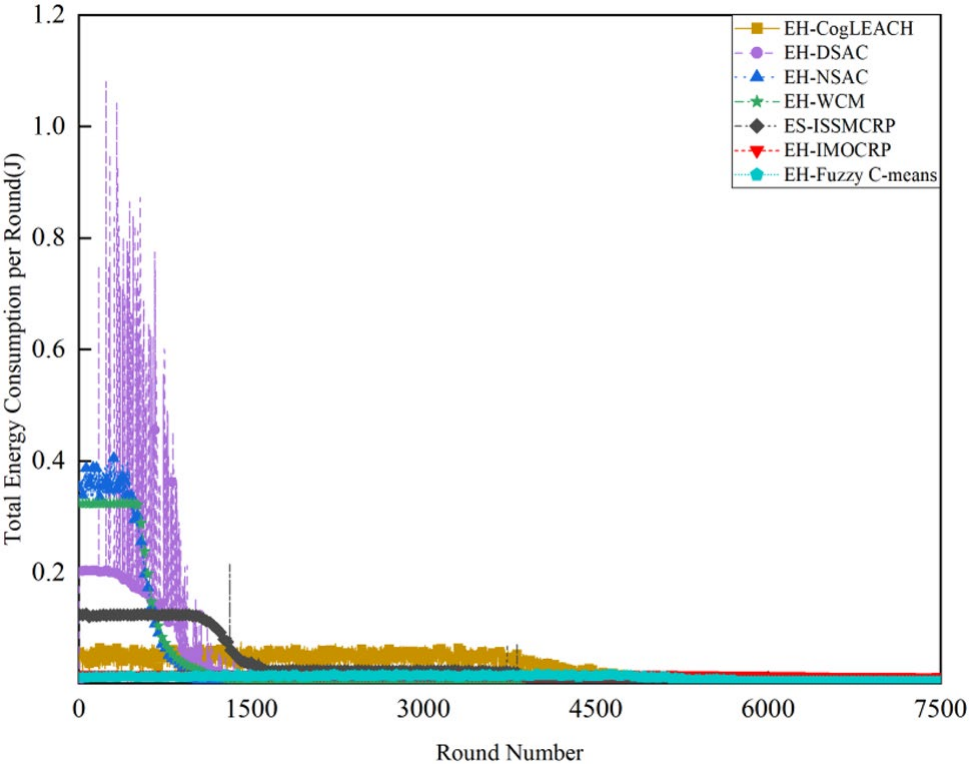
Comparison results of the total energy consumption per round across various protocols.

**Figure 6:**
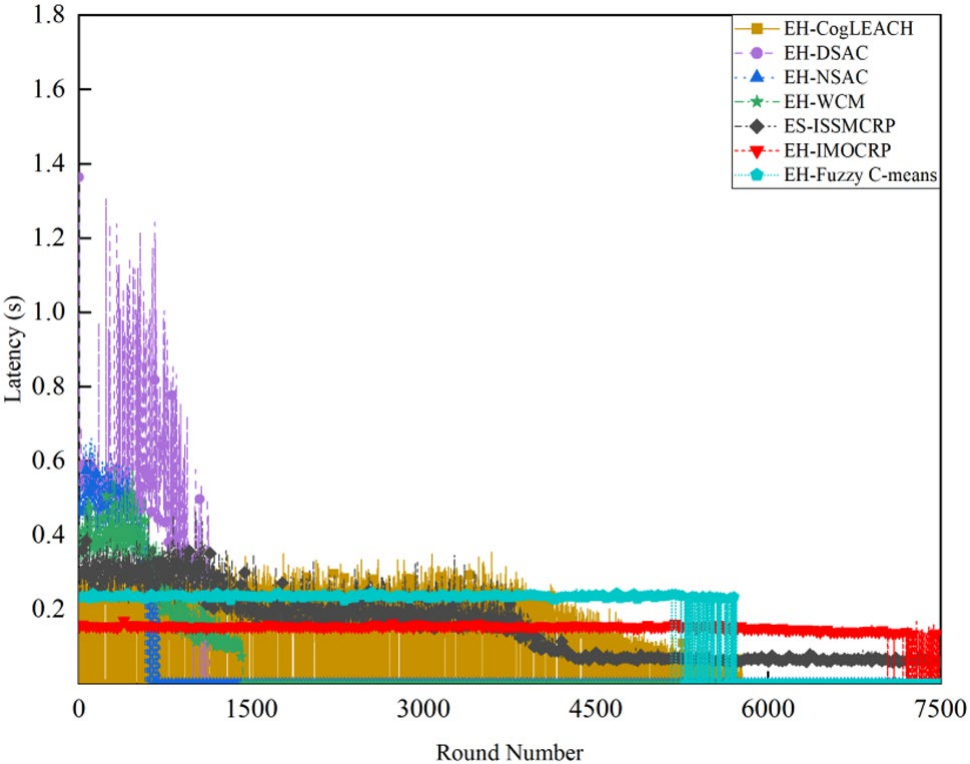
Comparison results of the latency across various protocols.

Figures 2 and 3 clearly show that ES-ISSMCRP protocol outperforms EH-DSAC, EH-NSAC, and EH-WCM protocols, but is inferior to EH-CogLEACH, EH-Fuzzy C-means, and EH-IMOCRP in terms of the number of surviving nodes. Additionally, ES-ISSMCRP achieves the highest performance in terms of the number of nodes effectively collecting data. It indicates that CRSNs nodes in ES-ISSMCRP consume less energy in control information exchange and data transmission. In order to explore the reasons behind this phenomenon, the total control overhead per round, the total energy consumption per round, and the latency are recorded in Figs. 4, 5 and 6. Here, the total control overhead per round is defined as the number of control packets exchanged during network operation per round, such as for CHs election, cluster formation, inter-cluster route establishment, and data transmission scheduling. The total network energy consumption per round refers to the aggregate energy consumed by all surviving nodes in each round, which includes the energy spent on control information exchange and data transmission. However, the energy consumption for spectrum sensing is not taken into consideration, as according to reference^8^, the energy consumed by each surviving node for spectrum sensing at the beginning of each round depends on the number of channels sensed, the duration of spectrum sensing, and the sampling frequency, etc. Once these parameters are fixed, the energy consumption for spectrum sensing is constant. As all surviving nodes in the comparative protocols incur this energy cost during the spectrum sensing stage, we have omitted the energy consumption for spectrum sensing in our simulations, ensuring fairness in our comparison results. Additionally, the energy consumption caused by data packet collisions is not considered because: (1) ES-ISSMCRP protocol primarily focuses on solving the network performance degradation problem due to imperfect spectrum sensing, mainly considering conflicts with PUs rather than packet collisions between various CHs during inter-cluster data transmission using the carrier sense multiple access/collision avoidance (CSMA/CA)approach. (2) Whether collisions occur and the number of retransmissions caused by these collisions during CSMA/CA channel access by CHs are random, and the corresponding energy consumption cannot be accurately calculated. Since such energy consumption exists in the comparative protocols as well, to ensure a fair comparison, we have not considered the additional energy consumption due to data packet collisions in simulations. The latency is defined as the maximum total delay introduced by spectrum sensing, CHs election, cluster formation, inter-cluster route establishment, intra-cluster data transfer, and multi-hop inter-cluster data relay. More details can be found in reference ^8^.

**Figure 7:**
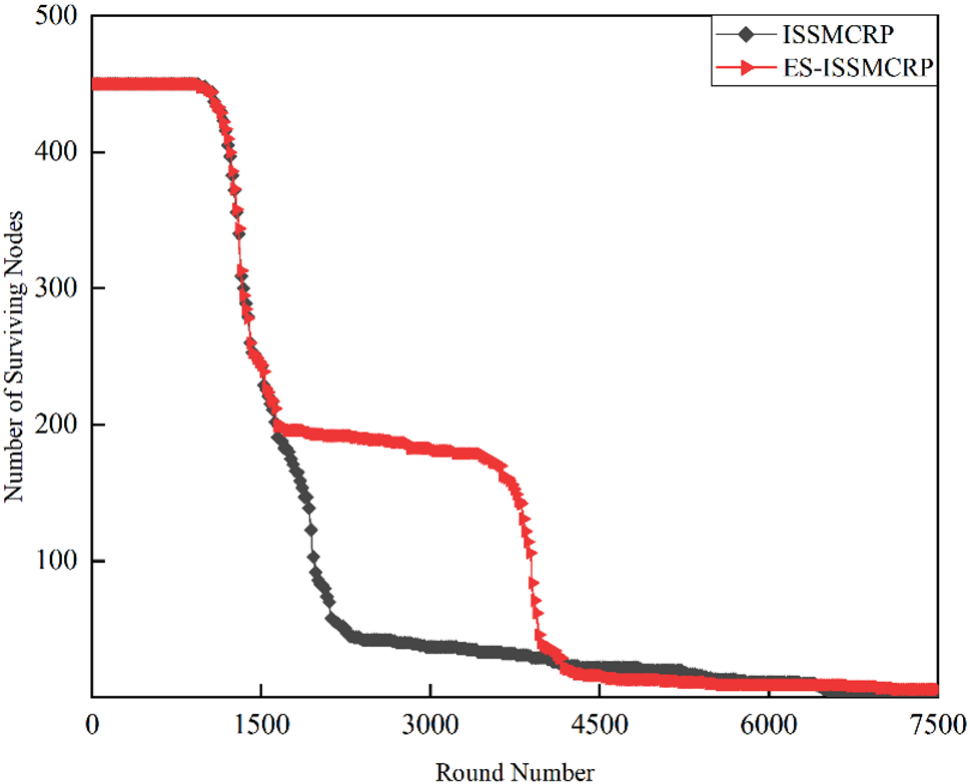
Comparisons between ES-ISSMCRP and ISSMCRP in terms of number of surviving nodes.

**Figure 8:**
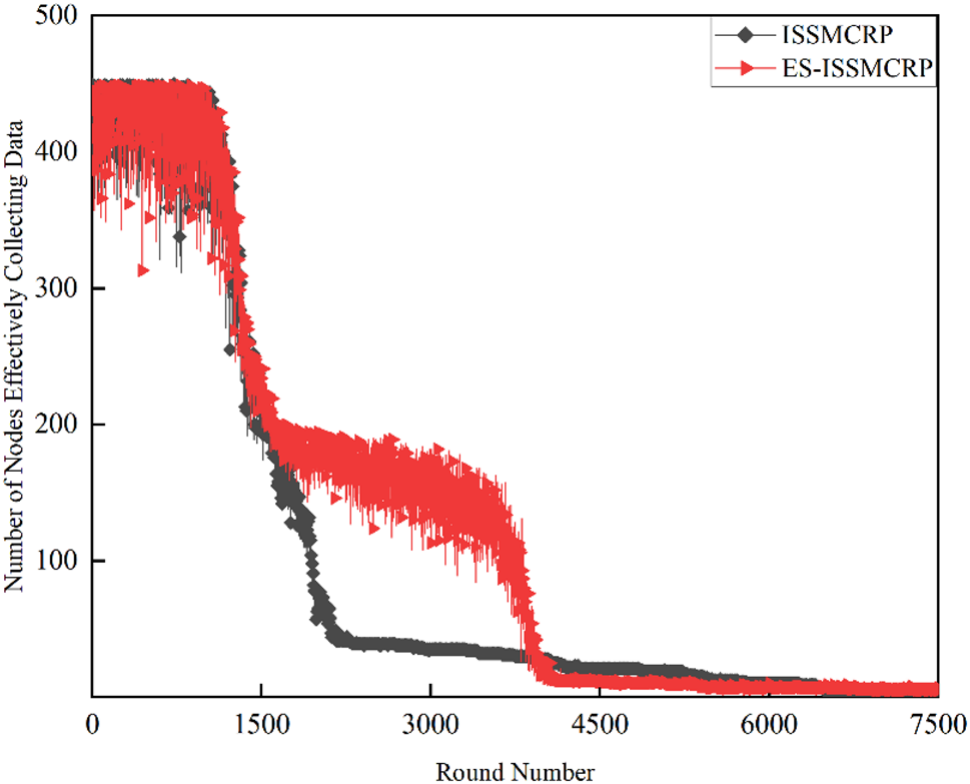
Comparisons between ES-ISSMCRP and ISSMCRP in terms of number of nodes effectively collecting data.

A detailed analysis of this superior performance is as follows:

1. ES-ISSMCRP protocol rigorously minimizes the control overhead and energy expenditure incurred during the CHs election and cluster formation to conserve the finite battery energy of CRSNs nodes. Specifically, CRSNs nodes in the first layer become independent CHs directly, eliminating the need for broadcasting CHs competition weights and thus avoiding the associated control overhead. Furthermore, during the CHs election process, CRSNs nodes beyond the first layer broadcast their own information and CHs competition weights to their neighbors. Neighboring nodes determine their potential to become CHs based on the received information. Nodes that qualify as CHs then disseminate CHs announcement messages, prompting ordinary nodes that receive these announcements to broadcast their withdrawal from the CHs competition. During cluster formation, nodes not designated as CHs request to join the cluster whose CH shares common idle channels and possesses the highest competition weight, which records these join requests. Therefore, the overall control overhead incurred during the CHs election and cluster formation for ES-ISSMCRP is more than triple but less than four times the number of surviving nodes. EH-WCM protocol incurs a total control overhead for CHs selection and cluster formation that is roughly four times the count of active nodes: all CRSNs nodes broadcast their spectrum sensing results and CHs weights on common control channel (CCC) to select CHs; neighboring nodes that receive this information decide if they are eligible to become CHs, with qualifying nodes broadcasting CHs announcements, and other nodes broadcasting their withdrawal from CHs competition; nodes not designated as CHs request to join the CH with the greatest weight and shared available channels, after which the CH communicates the cluster details to the sink. In EH-DSAC protocol, each CRSNs node starts as a CH and merges with adjacent clusters based on the common available channels and inter-cluster distances until they reach the optimal number derived from theoretical calculations. This involves substantial control information exchanges between CMs and CHs as well as among neighboring CHs, resulting in considerable energy expenditure, as indicated by Fig. 5. In EH-NSAC protocol, all CRSNs nodes calculate their own weight based on the remaining energy and channel quality, continuously update and broadcast their weight information. The node with the highest weight in the vicinity becomes a CH, with neighboring nodes joining to become CMs; unclustered nodes repeat this until clustering is completed. This process necessitates extensive control information exchanges among adjacent nodes, leading to significant energy usage. As a result, the first node death occurs earlier in EH-WCM, EH-DSAC, and EH-NSAC protocols than in ES-ISSMCRP protocol, with a sharp decline in the number of active nodes in subsequent rounds. EH-Fuzzy C-means and EH-IMOCRP represent centralized, single-hop clustering routing protocols for CRSNs. These protocols mandate that each surviving CRSNs node transmits information such as remaining energy to the sink. The sink is responsible for choosing CHs and communicating the clustering results to all CRSNs nodes. Consequently, their total control overhead per round is equivalent to the count of surviving nodes, as shown in Fig. 4. Therefore, their network lifespan is the longest among all comparative protocols. The first node death in EH-CogLEACH happens later than that in ES-ISSMCRP. This is due to the fact that the overhead for CHs selection and cluster formation in EH-CogLEACH is roughly twice the number of active nodes, which is relatively modest. In EH-CogLEACH, each CH broadcasts temporary and final CHs announcements, and ordinary nodes send temporary join requests and final confirmation to their CH. Despite lower energy usage under EH-CogLEACH, EH-Fuzzy C-means, and EH-IMOCRP, restriction to single-hop communication with the sink substantially restricts the network scalability and surveillance capacity. Additionally, while building clusters, EH-Fuzzy C-means fails to consider channel availability. Random channel selection may lead to the absence of a common channel between CMs and their CH, severely impacting the successful delivery of data packets. Therefore, the number of nodes effectively collecting data in EH-Fuzzy C-means is low. According to calculations, the average packet delivery ratio of ES-ISSMCRP protocol is 0.8209, while those of EH-CogLEACH, EH-Fuzzy C-means, and EH-IMOCRP protocols are only 0.0509, 0.0355, and 0.0814, respectively. In a word, although EH-CogLEACH, EH-Fuzzy C-means, and EH-IMOCRP have more surviving nodes, only a small proportion of these nodes are effective in data collection. Table 3 shows the average packet delivery ratios for the comparative protocols.
2. According to the aforementioned analysis, the total control overhead per round of ES-ISSMCRP is close to that of EH-WCM. However, as shown in Fig. 5, its total energy consumption per round is much lower than that of EH-WCM, EH-NSAC, and EH-DSAC. This is because nodes under ES-ISSMCRP exchange control information within cluster radius during CHs election and cluster formation stage, while nodes in other competing protocols exchange information within *R*_*t*_. Since the cluster radius is smaller than *R*_*t*_, nodes consume less energy in CHs election and cluster formation. Moreover, in order to make full use of the direct communication between CHs in layer 1 and the sink, in ES-ISSMCRP, CHs in layer 1 send their state messages directly to the sink in inter-cluster route establishment stage. The sink receives, aggregates, and broadcasts the message, which can reduce the number of control messages received by CHs in layer 2 and the energy consumption of competing for accessing CCC in layer 1. Thus, node energy is saved, and the network lifetime is prolonged.
3. As a multi-hop clustering routing protocol, EH-DSAC initially enables a larger count of nodes to route data to the sink through multiple hops, resulting in a higher number of nodes effectively collecting data. However, substantial control overhead leads to rapid energy depletion of nodes, with a marked decline in the number of surviving nodes and nodes effectively collecting data after round 618. EH-CogLEACH, EH-WCM, EH-NSAC, EH-Fuzzy C-means, and EH-IMOCRP protocols function as single-hop CRSNs clustering routing protocols, limiting effective data collection to nodes that can reach the sink in a single hop. Furthermore, the number of nodes effectively collecting data in EH-WCM and EH-NSAC protocols drastically decreases along with the swift reduction in the number of surviving nodes. ES-ISSMCRP protocol is a multi-hop clustering routing protocol that enables data to be transmitted to the sink through multi-hop routing. It also selects relatively stable channels with the highest idle detection accuracy for intra-cluster and inter-cluster data transmission, leading to infrequent channel reclaim by PUs.
4. From Fig. 6, we can observe that the latency of EH-DSAC is the longest among all competing protocols. This results from its excessive control information exchange for cluster merging and multi-hop route selection. More competing nodes within *R*_*t*_ increases the time required for successful channel access. ES-ISSMCRP can reduce the time required for control information exchange by controlling the cluster radius, but more effective data gathering nodes will inevitably increase the latency of data transmission. This is the price to pay for guaranteeing powerful network surveillance capability. Nonetheless, data packets in ES-ISSMCRP can still reach the sink within the round time.
5. By controlling cluster radii and implementing uneven clustering throughout the network, ES-ISSMCRP protocol reserves energy for relaying data packets from outer layers and equalizes the residual energy among CHs, ultimately extending network lifespan. Additionally, in ES-ISSMCRP protocol, the introduction of SWIPT technology for intra-cluster data transmission aids in further equalizing the residual energy between nodes within a cluster and extending the network lifespan. To confirm the effectiveness of intra-cluster SWIPT technology, this paper conducts a comparative analysis with ISSMCRP protocol, which does not utilize intra-cluster SWIPT technology. The detailed outcomes of this comparison are presented in Figs. 7 and 8.

**Table 3:** Average packet delivery ratios for the comparative protocols.

Protocols	Average packet delivery ratio
EH-CogLEACH	0.0509
EH-DSAC	0.2631
EH-NSAC	0.0688
EH-WCM	0.0479
EH-IMOCRP	0.0814
EH-Fuzzy C-means	0.0355
ES-ISSMCRP	0.8209

As illustrated in Figs. 7 and 8, during the periods before round 1413 and after round 4208, ES-ISSMCRP protocol demonstrates a similar number of surviving nodes and effective data-collecting nodes as ISSMCRP protocol. However, from round 1414 to 4207, ES-ISSMCRP protocol significantly surpasses ISSMCRP protocol in terms of both surviving nodes and nodes effectively collecting data, with an average increase of 102 surviving nodes and 80 nodes collecting data per round. In general, compared to ISSMCRP protocol, ES-ISSMCRP protocol achieves a 33.1% increase in surviving nodes and a 27.8% increase in effectively collecting data nodes, thereby confirming the effectiveness of the implementation of intra-cluster SWIPT technology in ES-ISSMCRP protocol.

**Figure 9:**
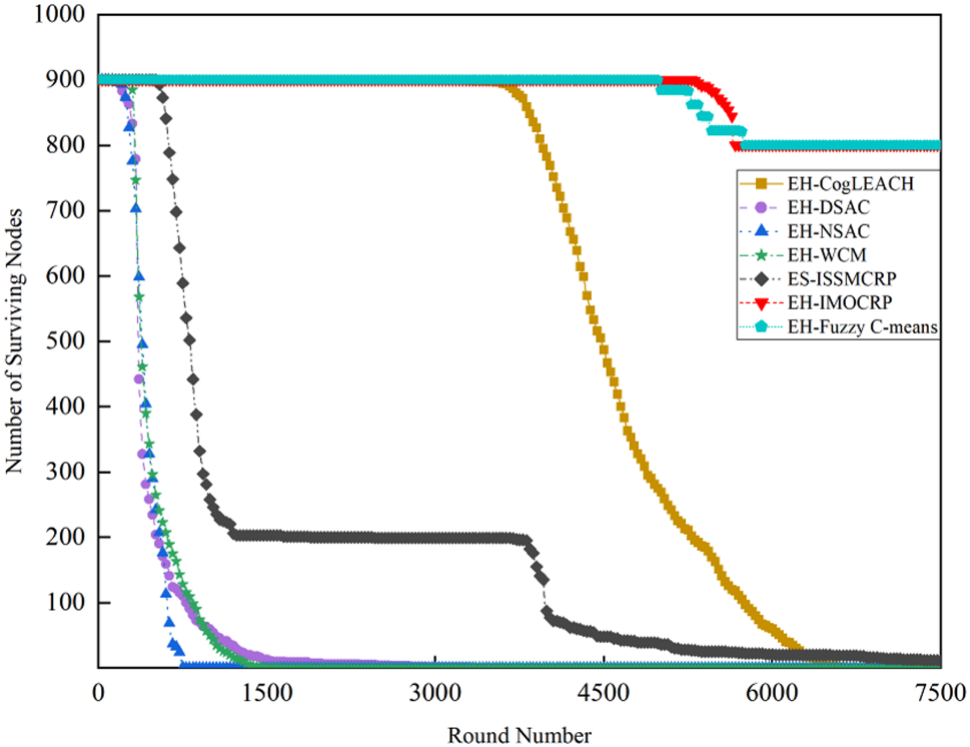
Comparison results of the number of surviving nodes in 900-node CRSNs.

**Figure 10:**
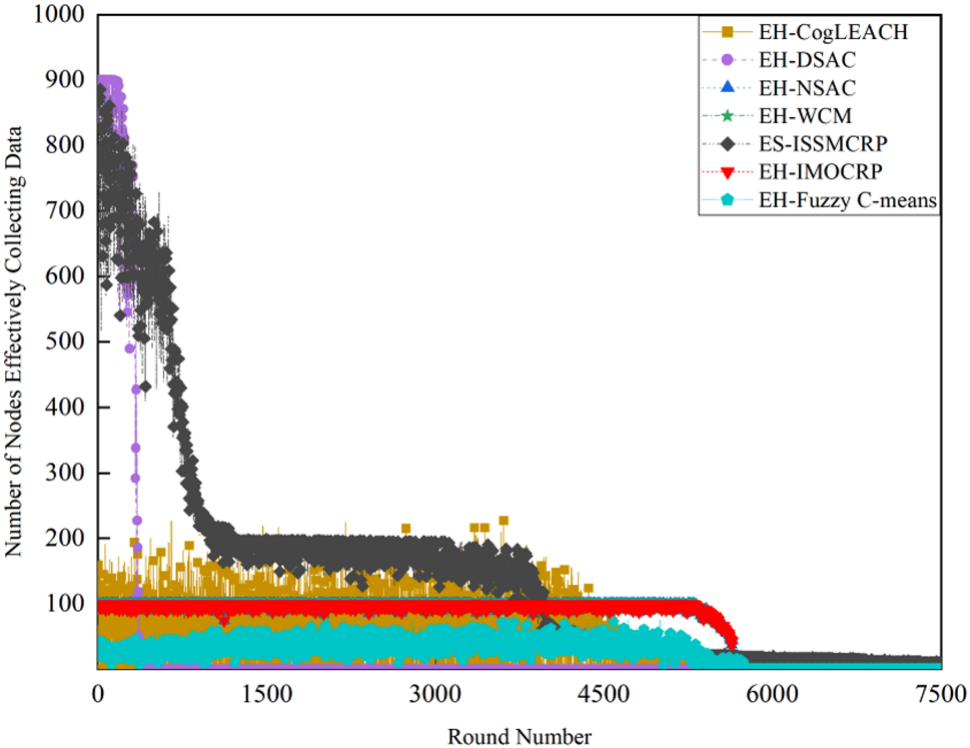
Comparison results of the number of nodes effectively collecting data in 900-node CRSNs.

**Figure 11:**
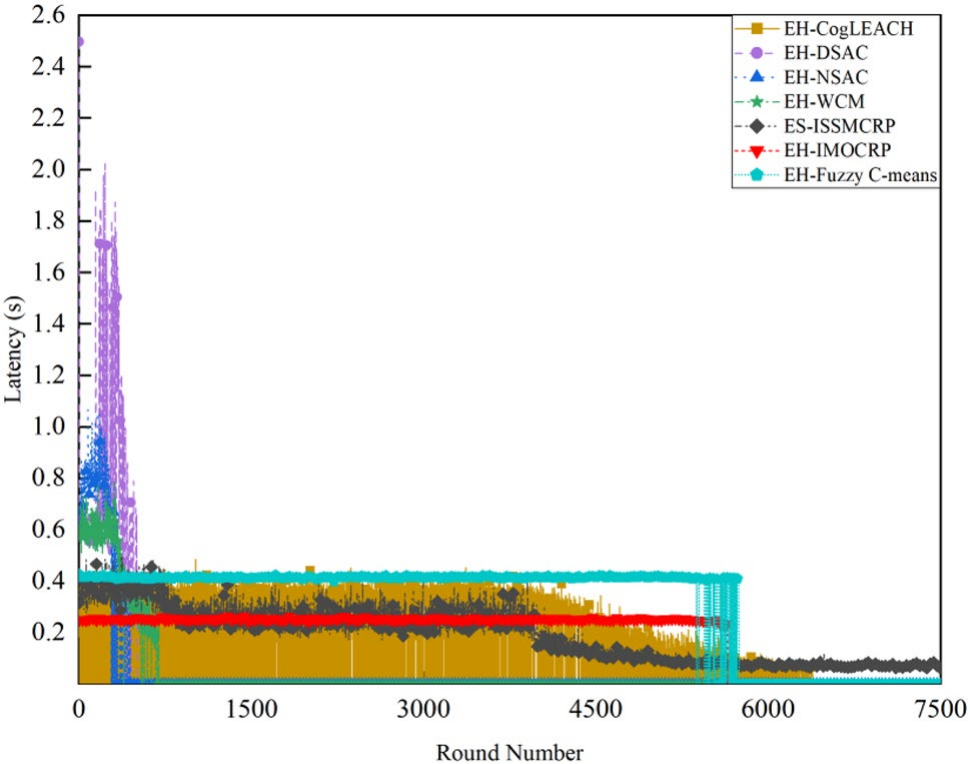
Comparison results of the latency in 900-node CRSNs.

While keeping the network radius *R* unchanged, as the number of CRSNs nodes increases from 450 to 900, the simulation results for the number of surviving nodes, nodes effectively collecting data, and latency under various clustering protocols are illustrated in Figs. 9, 10 and 11.

A comparison with the simulation results for 450-node CRSNs reveals that the increase in node density *ρ* does not significantly affect the number of surviving nodes in single-hop clustering protocols such as EH-CogLEACH, EH-Fuzzy C-means, and EH-IMOCRP, but there are noticeable changes in the number of nodes effectively collecting data and in latency. Specifically, the number of nodes effectively collecting data in EH-CogLEACH and EH-IMOCRP protocols are approximately doubled, while the increase in EH-Fuzzy C-means is under two-fold. The primary reason for this phenomenon is analyzed below: all CRSNs nodes are randomly and uniformly distributed, and when *ρ* doubles, the number of nodes that can reach the sink in one hop also doubles. As all these protocols are single-hop clustering protocols, only nodes that can reach the sink in a single hop can transmit data to it. However, due to the effects of random channel selection, the number of nodes effectively collecting data under EH-Fuzzy C-means protocol is noticeably lower than the number of nodes that can reach the sink in one hop, consistent with the preceding theoretical analysis. The significant increase in the latency is mainly due to the fact that when *ρ* doubles, the number of competing nodes within the maximum transmission range *R*_*t*_ increases, leading to greater delays in nodes accessing CCC to exchange control information. Additionally, an increase in the number of clusters or the number of nodes within a cluster leads to increased delays in intra-cluster data aggregation or inter-cluster data transmission. The first node death in the proposed ES-ISSMCRP protocol occurs earlier than in the 450-node CRSNs. The reason behind this phenomenon lies in the fact that increasing *ρ* leads to a higher total number of clusters formed. An increased number of data packets need to be relayed towards the sink, which will cause higher energy consumption for inner-layer nodes. Although ES-ISSMCRP can balance the energy distribution among nodes in different layers, it cannot entirely avoid the network lifespan reduction caused by heavier data relay. The number of nodes effectively collecting data in ES-ISSMCRP protocol generally shows a linear relationship with the number of surviving nodes, ensuring sustained high network surveillance performance throughout an extended network lifetime. The average packet delivery ratio of ES-ISSMCRP protocol is calculated to be 0.8266. Furthermore, by stringently regulating the cluster radius, ES-ISSMCRP protocol efficiently controls the number of nodes competing for channel access and the number within each cluster, leading to a marginal increase in latency. However, the impact of the increased node density is not pronounced.

The proposed ES-ISSMCRP protocol can be applied in EH-CRSNs to supplement and balance the remaining energy among nodes, thereby extending the network lifespan while maintaining powerful network surveillance capabilities. For instance, it can be used in the field of agricultural production, assisting in obtaining information regarding crops, plants, temperature measurement, humidity, and irrigation systems to facilitate precision and automated agriculture^40^. It can also be utilized in health monitoring, aiding in the collection of physiological parameters to advance medical informatics^41^. Additionally, it can be used for continuous monitoring of forest fires, enabling timely detection of fires to prevent significant loss of lives, wildlife, and property^42^.

## Conclusions

Building on our previously proposed ISSMCRP protocol, this paper introduces an SWIPT-based multi-hop clustering routing protocol ES-ISSMCRP for EH-CRSNs. ES-ISSMCRP protocol effectively leverages downlink RF EH and SWIPT technologies to enhance and equalize the remaining energy among nodes, substantially prolonging the network lifespan while maintaining its surveillance capabilities. Simulation results show that ES-ISSMCRP protocol achieves notable improvements compared to ISSMCRP protocol. Specifically, under ES-ISSMCRP protocol, there is a 33.1% increase in network lifespan and a 27.8% enhancement in surveillance capabilities. When compared to other protocols, ES-ISSMCRP protocol features the combined strengths of extending network lifespan and improving surveillance capabilities. In particular, it extends network lifespan by 88.1% compared to EH-DSAC, EH-NSAC, and EH-WCM protocols, and increases surveillance capability by 83.5% compared to EH-CogLEACH, EH-Fuzzy C-means, and EH-IMOCRP protocols. Nonetheless, within ES-ISSMCRP protocol, EH-CRSNs nodes gather energy solely through direct links with the sink, and significant path loss reduces EH efficiency for nodes distant from the sink, limiting the potential of EH technology in extending the overall network lifespan. To address this, future plans involve incorporating intelligent reflecting surface (IRS) in CRSNs, allowing signals from both direct links and IRS-cascaded reflection links to coherently combine at the receiver, thus increasing the energy harvested and enhancing EH efficiency, leading to an extended network lifespan. Additionally, as a time-triggered clustering routing protocol for CRSNs, ES-ISSMCRP protocol is not capable of simultaneously satisfying the distinguished requirements of event-driven traffic. To avoid the necessity for each CRSNs node to run multiple clustering protocols to serve different types of traffic, based on our previous work^43^, we plan to evolve ES-ISSMCRP protocol into a traffic-driven clustering routing protocol, capable of supporting both time-triggered and event-driven traffic.

## Data Availability

The datasets generated during and/or analyzed during the current study are available from the corresponding author on reasonable request.

## References

[1] Wang JH, Li S (2021). ECE: A novel performance evaluation metric for clustering protocols in cognitive radio sensor networks. IEEE Internet Things J..

[2] Liu ZX, Zhao MY, Yuan YZ, Guan XP (2020). Subchannel and resource allocation in cognitive radio sensor network with wireless energy harvesting. Comput. Netw..

[3] Zareei M (2019). Enhancing the performance of energy harvesting sensor networks for environmental monitoring applications. Energies.

[4] Misra, S. & Kumar, R. A literature survey on various clustering approaches in wireless sensor network. In *2016 2nd International Conference on Communication Control and Intelligent Systems* (*CCIS*), 18–22 (2016).

[5] Hector K, Karel T, Jorge TG (2021). Energy-efficient cooperative spectrum sensing based on stochastic programming in dynamic cognitive radio sensor networks. IEEE Access..

[6] Shavhov V, Koo I (2021). An efficient clustering protocol for cognitive radio sensor networks. Electronics.

[7] Ye HY, Jiang JB (2021). Optimal linear weighted cooperative spectrum sensing for clustered-based cognitive radio networks. EURASIP J. Wirel. Commun. Network..

[8] Wang JH, Ge YY (2022). A radio frequency energy harvesting-based multihop clustering routing protocol for cognitive radio sensor networks. IEEE Sens. J..

[9] Nundakwang, S., Yingyong, P. & Isarakorn, D. Energy harvesting for self-powered systems. In *2020 6th International Conference on Engineering, Applied Sciences and Technology* (*ICEAST*), 1–4 (2020).

[10] Lu X, Wang P, Niyato D, Hossain E (2014). Dynamic spectrum access in cognitive radio networks with RF energy harvesting. IEEE Wirel. Commun..

[11] Prajapat R, Yadav RN, Misra R (2021). Energy-efficient k-hop clustering in cognitive radio sensor network for internet of things. IEEE Internet Things J..

[12] El Shafie A, Khattab T, El-Keyi A, Nafie M (2016). On the coexistence of a primary user with an energy harvesting secondary user: A case of cognitive cooperation. Wirel. Commun. Mobile Comput..

[13] Latiwesh, A. & Dong, Y. Q. Energy efficient spectrum aware clustering for cognitive sensor networks: CogLEACH-C. In *2015 10th International Conference on Communications and Networking in China* (*ChinaCom*), 515–520 (2015).

[14] Bhatti DMS, Saeed N, Nam H (2016). Fuzzy C-means clustering and energy efficient cluster head selection for cooperative sensor network. Sensors.

[15] Wang JH, Li S, Ge YY (2020). Ions motion optimization-based clustering routing protocol for cognitive radio sensor network. IEEE Access..

[16] Eletreby, R. M., Elsayed, H. M., Khairy, M. M. CogLEACH: A spectrum aware clustering protocol for cognitive radio sensor networks. In *2014 9th International Conference on Cognitive Radio Oriented Wireless Networks and Communications* (*CROWNCOM*), 179–184 (2014).

[17] Zheng M, Chen S, Liang W, Wang CQ (2018). Network stability-aware clustering protocol for cognitive radio sensor networks. J. Softw..

[18] Wang TJ, Guan XJ, Wan XL, Shen H, Zhu XM (2019). A spectrum-aware clustering algorithm based on weighted clustering metric in cognitive radio sensor networks. IEEE Access..

[19] Zhang, H. Z., Zhang, Z. Y., Dai, H. Y., Yin, R. & Chen, X. M. Distributed spectrum-aware clustering in cognitive radio sensor networks. In *2011 IEEE Global Telecommunications Conference—GLOBECOM 2011*, 1–6 (2011).

[20] Yadav RN, Misra R, Saini D (2018). Energy aware cluster based routing protocol over distributed cognitive radio sensor network. Comput. Commun..

[21] Zheng M, Wang CQ, Song M, Liang W, Yu HB (2021). SACR: A stability-aware cluster-based routing protocol for cognitive radio sensor networks. IEEE Sens. J..

[22] Pei ER, Han HZ, Sun ZH, Shen B, Zhang TQ (2015). LEAUCH: Low-energy adaptive uneven clustering hierarchy for cognitive radio sensor network. EURASIP J. Wirel. Commun. Network..

[23] Zhang MC, Zheng RJ, Li Y, Wu QT, Song L (2016). R-bUCRP: A novel reputation-based uneven clustering routing protocol for cognitive wireless sensor networks. J. Sens..

[24] Wang CQ, Wang SB (2019). Research on uneven clustering APTEEN in CWSN based on ant colony algorithm. IEEE Access..

[25] Stephan T, Al-Turjman F, Suresh JK, Balusamy B (2021). Energy and spectrum aware unequal clustering with deep learning based primary user classification in cognitive radio sensor networks. Int. J. Mach. Learn. Cybern..

[26] Wang JH, Liu C (2023). An imperfect spectrum sensing-based multi-hop clustering routing protocol for cognitive radio sensor networks. Sci. Rep..

[27] Wang JH, Li CH (2022). An energy balance-oriented clustering routing protocol for cognitive radio sensor networks. IEEE Sens. J..

[28] Gurjar DS, Nguyen HH, Pattanayak P (2019). Performance of wireless powered cognitive radio sensor networks with nonlinear energy harvester. IEEE Sens. Lett..

[29] Talukdar, B., Kumar, D. & Arif, W. Performance analysis of a SWIPT enabled cognitive radio sensor network using TS protocol. In *2020 Advanced Communication Technologies and Signal Processing* (*ACTS*)*, *1–5 (2020).

[30] Kumar D, Singya PK, Choi K, Bhatia V (2023). SWIPT enabled cooperative cognitive radio sensor network with non-linear power amplifier. IEEE Trans. Cognit. Commun. Network..

[31] Kang JM, Kim IM, Kim DI (2018). Wireless information and power transfer: Rate-energy tradeoff for nonlinear energy harvesting. IEEE Trans. Wirel. Commun..

[32] Motamedi, A. & Bahai, A. MAC protocol design for spectrum-agile wireless networks: Stochastic control approach. In *2007 2nd IEEE International Symposium on New Frontiers in Dynamic Spectrum Access Networks*, 448–451 (2007).

[33] Ali A, Hamouda W (2017). Advances on spectrum sensing for cognitive radio networks: Theory and applications. IEEE Commun. Surv. Tutor..

[34] Wu H, Yao FQ, Chen Y, Liu YX, Liang T (2017). Cluster-based energy efficient collaborative spectrum sensing for cognitive sensor network. IEEE Commun. Lett..

[35] Zahabi SJ, Tadaion AA, Aissa S (2012). Neyman-pearson cooperative spectrum sensing for cognitive radio networks with fine quantization at local sensors. IEEE Trans. Commun..

[36] Lu X, Wang P, Niyato D, Kim DI, Han Z (2015). Wireless networks with RF energy harvesting: A contemporary survey. IEEE Commun. Surv. Tutor..

[37] Chen Y, Sabnis KT, Abd-Alhameed RA (2016). New formula for conversion efficiency of RF EH and its wireless applications. IEEE Trans. Veh. Technol..

[38] Heinzelman WB, Chandrakasan AP, Balakrishnan H (2002). An application-specific protocol architecture for wireless microsensor networks. IEEE Trans. Wirel. Commun..

[39] Wang JH, Liu C (2022). Nonlinear energy harvesting-based available energy evolution model for cognitive radio sensor networks. J. Network Intell..

[40] Haseeb K, Din IU, Almogren A, Islam N (2020). An energy efficient and secure IoT-based WSN framework: An application to smart agriculture. Sensors.

[41] Amjad O, Bedeer E, Ikki S (2019). Energy-efficiency maximization of self-sustained wireless body area sensor networks. IEEE Sens. Lett..

[42] Abdullah, S., Bertalan, S., Masar, S., Coskun, A. & Kale, I. A wireless sensor network for early forest fire detection and monitoring as a decision factor in the context of a complex integrated emergency response system. *In 2017 IEEE Workshop on Environmental, Energy, and Structural Monitoring Systems (EESMS)*, 1–5 (2017).

[43] Wang JH, Ni H, Ge YY, Li S (2022). Traffic-driven ions motion optimization-based clustering routing protocol for cognitive radio sensor networks. PLoS One.

